# Prognostic Role of Neutrophil to Lymphocyte Ratio in Nonalcoholic Fatty Liver Disease: A Systematic Review and Meta-Analysis

**DOI:** 10.1155/2022/1554079

**Published:** 2022-09-10

**Authors:** Mitra Shavakhi, Shima Nourigheimasi, Emma Dioso, Michael Goutnik, Brandon Lucke-Wold, Shokoufeh Khanzadeh, Fariba Heidari

**Affiliations:** ^1^Department of Pathology, School of Medicine, Isfahan University of Medical Sciences, Iran; ^2^School of Medicine, Arak University of Medical Sciences, Arak, Iran; ^3^Department of Neurosurgery, University of Utah, Salt Lake City, USA; ^4^Department of Neurosurgery, University of Florida, Gainesville, USA; ^5^Student Research Committee, Tabriz University of Medical Sciences, Tabriz, Iran; ^6^Department of Community and Family Medicine, Faculty of Medicine, Tabriz University of Medical Sciences, Tabriz, Iran

## Abstract

**Introduction:**

Nonalcoholic steatohepatitis (NASH) and liver fibrosis are the most common complications of nonalcoholic fatty liver disease (NAFLD). In this systematic review and meta-analysis, we aim to analyze the current literature to evaluate the association of neutrophil to lymphocyte ratio (NLR) with NASH and fibrosis in patients with NAFLD.

**Methods:**

PubMed, Web of Science, and Scopus were used to conduct a systematic search for relevant publications published before May 24, 2022. The Newcastle–Ottawa scale was used for quality assessment.

**Results:**

Thirteen studies were included in our study. The pooled results showed that NAFLD patients with significant NASH had elevated levels of NLR compared to those with nonsignificant or without NASH (SMD = 0.97, 95% CI = 0.59–1.39, *p* < 0.001). The pooled sensitivity and specificity of NLR were 78.16% (95% CI = 73.70%–82.04%), and 76.93% (95% CI = 70.22%–82.50%), respectively. In addition, NAFLD patients with significant liver fibrosis had elevated levels of NLR compared to those with nonsignificant or without fibrosis (SMD = 1.59, 95% CI = 0.76–2.43, *p* < 0.001). The pooled sensitivity and specificity of NLR were 82.62% (95% CI = 70.235%–90.55%) and 81.22% (95% CI = 75.62%–85.78%), respectively.

**Conclusion:**

Our findings support NLR to be a promising biomarker that can be readily integrated into clinical settings to aid in the prediction and prevention of NASH and fibrosis among patients with NAFLD.

## 1. Introduction

Nonalcoholic fatty liver disease (NAFLD) is an increasingly prevalent (approximately 25% global prevalence) [1] clinical disease that is associated with obesity, type II diabetes, and other metabolic comorbidities that are increasingly common in modern society. [2] Nonalcoholic steatohepatitis (NASH) and liver fibrosis are the most common complications of NAFLD. In fact, NASH is a histological phenotype of NAFLD that represents a significant inflammatory progression from simple steatosis that may subsequently progress to cirrhosis and hepatocellular carcinoma and is becoming an increasingly common indication for liver transplantation. [2] The neutrophil to lymphocyte ratio (NLR) is an easily obtained serum measure that corresponds to systemic inflammation and has been demonstrated to be a useful prognostic measure in a variety of pathologies, including stroke and colorectal cancer. [3, 4] In several studies, NLR has been positively associated with NASH and fibrosis stage in patients with NAFLD [5–17]. Thus, NLR might serve as an easily obtainable predictive tool to guide clinical decision-making, intervene earlier and improve patient outcomes. The goal of this systematic review and meta-analysis is to analyze existing retrospective and prospective studies to establish the potential utility of NLR in the prediction of NASH and fibrosis among patients with NAFLD. To the best of our knowledge, this is the first meta-analysis in this context.

## 2. Materials and Methods

### 2.1. Study Design and Eligibility Criteria

This study is conducted according to the Preferred Reporting Items for Systematic Reviews and Meta-analyses (PRISMA) 2020 reporting guideline [18]. We searched databases of PubMed, Web of Science, and Scopus up to May 24, 2022. In our literature search, we used the following search strategy: (‘Neutrophil to lymphocyte ratio' or NLR) and (‘nonalcoholic fatty liver' or ‘nonalcoholic fatty liver' or NAFLD) and (steatohepatitis or NASH or fibrosis).

Additionally, we reviewed the reference lists of included and relevant studies to identify further eligible studies. Our inclusion criteria were based on the following PICO terms:

- Population: NAFLD patients with significant NASH of fibrosis.
- Intervention: NLR.
- Control: NAFLD patients with nonsignificant or without NASH or fibrosis.
- Outcomes: The diagnostic performance of NLR.
- Study Design: We expected papers to be case-control or cross-sectional. However, we did not limit our search to any particular research design.

Our exclusion criteria were as follows: (1) review articles, editorials/letters, case series, case reports, abstracts, and randomized controlled trials; (2) duplicate studies; (3) not peer-reviewed publications. There were not any limitations on language or date of publication.

### 2.2. Data Extraction and Quality Assessment

The first author, year of publication, study design, study location, total sample size, number of cases and controls, mean and SD of NLR level, or any data for estimating the mean and SD (median and IQR or/and range), a cut-off value of NLR and its false/true positive and false/true negative from 2 × 2 table were all extracted. When the number of patients in false/true positive and false/true groups was not reported, we calculated it using sensitivity, and specificity. Two authors conducted the quality assessment of included studies, based on the Newcastle–Ottawa scale (NOS), including three components selection of the cohort, comparability of cohorts based on the design or analysis, how the exposure was ascertained, and how the outcomes of interest were assessed [19]. Disagreements between the authors were finally resolved via consensus. Those studies with six or more points were deemed to have good quality.

### 2.3. Data Synthesis and Analysis

We performed the meta-analysis by using Stata 11.2 software (Stata Corp, College Station, TX). We used standardized mean difference (SMD) with a 95% confidence interval (CI) to compare the NLR level between cases and controls. The I^2^ and Cochran's *Q* tests were adopted to determine the heterogeneity of the included studies. Significant heterogeneity between studies was conceived as I^2^ > 50% and *p* value of the *Q* test < 0.05. Finally, because a significant level of heterogeneity was found, we applied the random-effects model to calculate pooled effects. In order to determine the diagnostic value of NLR for NASH or fibrosis, we used the “metandi” command which estimated pooled sensitivity, specificity, diagnostic odds ratio (DOR), negative likelihood ratio, and positive likelihood ratio. In addition, a summary receiver operating characteristic (SROC) curve was drawn. In order to determine the publication bias, we used the funnel plot and Egger test.

## 3. Results

### 3.1. Search and Selection of Literature

**Figure 1:**
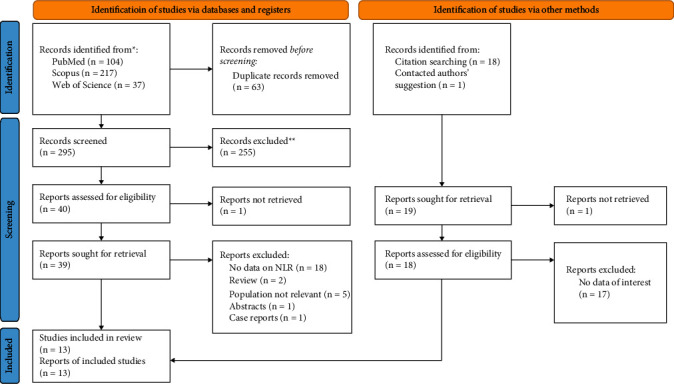
PRISMA 2020 Flow diagram for new systematic reviews which includes searches of databases, registers, and other sources.

The database search and manual search of the article citation list yielded a total of 377 results. Finally, 13 papers were included in this systematic review and meta-analysis [5–17]. Of them, 12 studies, including 893 cases and 1176 controls, compared the NLR level of NAFLD patients with significant NASH compared to those with nonsignificant or without NASH [5–16]. Of 12 studies, six studies reported the results of receiver operating characteristic (ROC) curve analysis, including the best cut-off point, sensitivity, and specificity of NLR in the prediction of NASH among NAFLD patients [5–7, 12, 14, 15]. In addition, seven studies, including 316 cases and 336 controls, compared the NLR level of NAFLD patients with significant fibrosis compared to those with nonsignificant or without fibrosis [5, 6, 11, 12, 14, 16, 17], and of them, four studies conducted receiver operating characteristic (ROC) curve analysis [5, 6, 12, 14]. The process of inclusion and exclusion is detailed in the PRISMA flow diagram, provided in Figure 1.

### 3.2. Characteristics of Included Studies

The characteristics and methodological qualities of these studies were shown in Table 1. The overall study quality ranged from 6 to 8 stars. Thirteen studies were included in our systematic review and meta-analysis. Three studies were retrospective and others were prospective. All of them were written in English.

### 3.3. Association of NLR and NASH among NAFLD Patients

**Figure 2:**
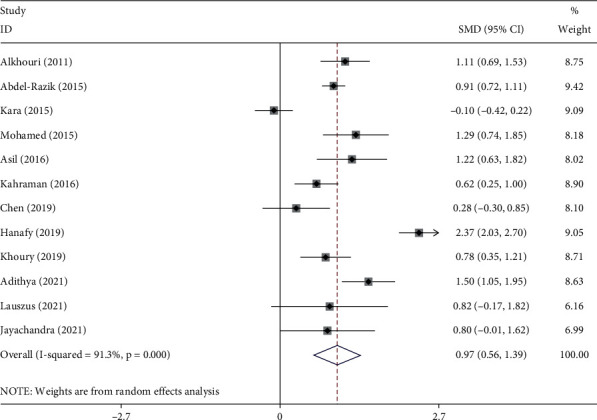
Meta-analysis of differences in NLR level between NAFLD patients with significant NASH compared to those with nonsignificant or without NASH.

The pooled results showed that NAFLD patients with significant NASH had elevated levels of NLR than those with nonsignificant or without NASH (SMD = 0.97, 95% CI = 0.59–1.39, *p* < 0.001, I^2^ = 91.3%, random-effects model) (Figure 2).

**Figure 3:**
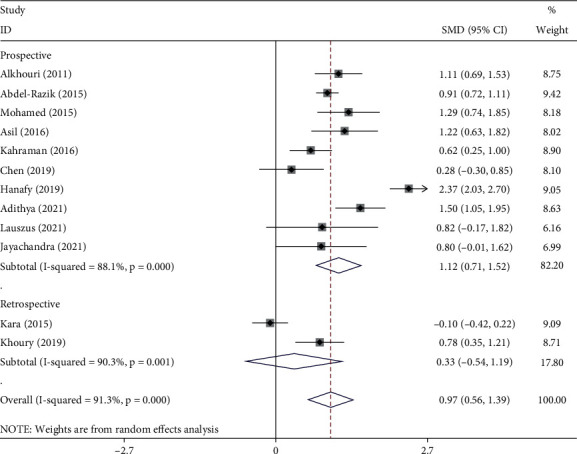
Subgroup analysis of differences in NLR level between NAFLD patients with significant NASH compared to those with nonsignificant or without NASH, according to study design.

In subgroup analysis according to study design, NAFLD patients with significant NASH had had elevated levels of NLR compared to those with nonsignificant or without NASH in prospective studies (SMD = 1.12, 95% CI = 0.71–1.52, *p* < 0.001) but not in retrospective studies (SMD = 0.33, 95% CI = −0.54–1.19, *p*=0.459) (Figure 3).

### 3.4. Diagnostic Value of NLR in NASH among NAFLD Patients

**Figure 4:**
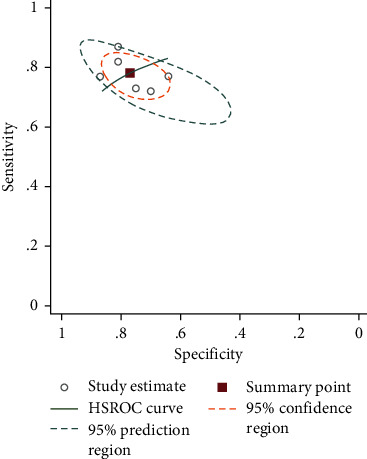
SROC curve of included studies in the meta-analysis of the association of NLR with NASH.

The pooled sensitivity of six studies was 78.16% (95% CI = 73.70%–82.04%), and the pooled specificity was 76.93% (95% CI = 70.22%–82.50%). The pooled positive likelihood ratio, negative likelihood ratio, and DOR of NLR were 3.38 (95%CI = 2.52–4.54), 0.28 (95%CI = 0.22–0.36), and 11.93 (95%CI = 7.19–19.78), respectively (Figure 4).

### 3.5. Association of NLR and Liver Fibrosis among NAFLD Patients

**Figure 5:**
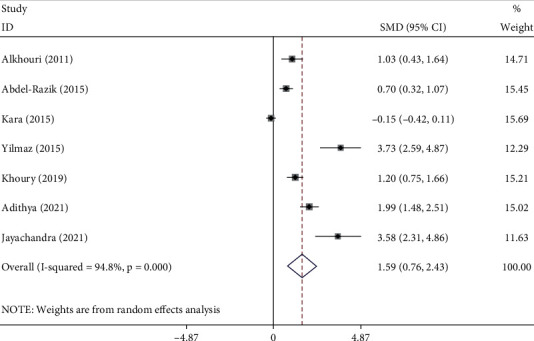
Meta-analysis of differences in NLR level between significant liver fibrosis compared to those with nonsignificant or without fibrosis.

As seen in Figure 5, NAFLD patients with significant liver fibrosis had had elevated levels of NLR than those with nonsignificant or without fibrosis (SMD = 1.59, 95% CI = 0.76–2.43, *p* < 0.001, I^2^ = 94.8%, random-effects model).

**Figure 6:**
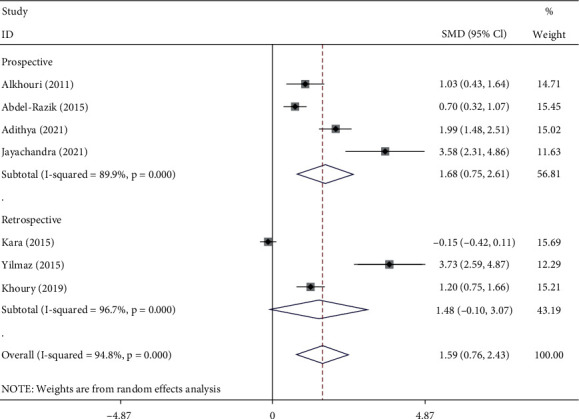
Subgroup analysis of differences in NLR level between NAFLD patients with significant fibrosis compared to those with nonsignificant or without fibrosis, according to study design.

In subgroup analysis according to study design, NAFLD patients with significant liver fibrosis had had elevated levels of NLR compared to those with nonsignificant or without fibrosis in prospective studies (SMD = 1.68, 95% CI = 0.75–2.61, *p* < 0.001) but not in retrospective studies (SMD = 1.48, 95% CI = −0.10–3.07, *p*=0.06) (Figure 6).

### 3.6. Diagnostic Value of NLR in Liver Fibrosis among NAFLD Patients

**Figure 7:**
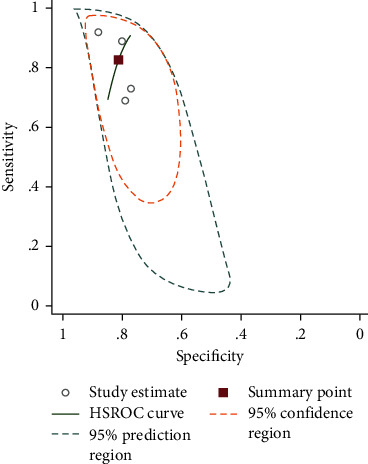
SROC curve of included studies in the meta-analysis of the association of NLR with fibrosis.

The pooled sensitivity of four studies was 82.62% (95% CI = 70.235%–90.55%), and the pooled specificity was 81.22% (95% CI = 75.62%–85.78%). The pooled positive likelihood ratio, negative likelihood ratio, DOR of NLR were 4.40 (95%CI = 3.08–6.28), 0.21 (95%CI = 0.11–0.39), and 20.58 (95%CI = 8.05–52.58), respectively (Figure 7).

### 3.7. Publication Bias

**Figure 8:**
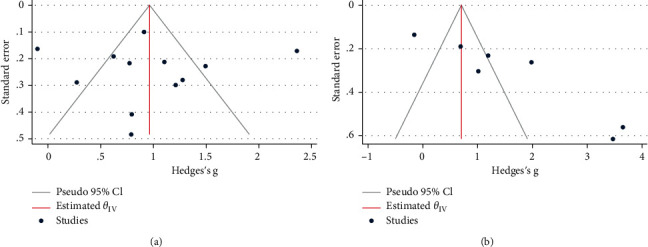
Funnel plots assessing publication bias. (a) studies on the usefulness of NLR for predicting NASH; (b) studies on the usefulness of NLR for predicting liver fibrosis.

As seen in Figure 8, there was some indication of publication bias among studies on the usefulness of NLR for the prediction of either NASH (Egger's test *p*=0.80, Begg's *p*=0.73) or liver fibrosis (Egger's test *p* < 0.001; Begg's test *p*=0.07) among NAFLD patients.

## 4. Discussion

In the presence of inflammatory disease, circulating neutrophils often increase, and circulating lymphocytes often decrease. [20] As inflammatory markers, neutrophils and lymphocytes may play multiple roles in the progression of chronic inflammatory diseases, including NAFLD. Currently, the progression of NAFLD is described as a ‘two-hit hypothesis.' [21] Initially, the ‘first hit' is defined as triglycerides accumulate in hepatocytes and insulin resistance develops as a hepatic manifestation. [21] This steatosis essentially desensitizes the liver to further inflammation, allowing the progression to NASH. [22] In 2011, Kamari and colleagues illustrated significant associations between increased NLR and insulin resistance pathologies using mouse models. [23] Notably, an overproduction of IL-1*α* and IL-1*β* from resident liver cells is observed in the development of NASH, as a deficiency in either interleukin was observed to sufficiently protect against NASH development. [23] Additionally, the lipotoxicity leading to the development of NASH kills hepatocytes through apoptosis and necrosis. [24] In turn, necrosis activates macrophages, neutrophils, and proinflammatory pathways, resulting in an elevated NLR. [24, 25].

The triglyceride accumulation and lipotoxicity are followed by the ‘second hit' activation of systemic proinflammatory pathways. Specifically, inflammatory cytokines (notably, IL-1, IL-6, and TNF-*α*), chemokines, chemokine receptors, adhesion molecules, and signal molecules are increased in both NASH animal models and clinical studies of NASH patients. [25] Farrell and colleagues describe nuclear factor-kappa B (NF-*κ*B) and c-Jun *N*-terminal kinase (JNK) as the key proinflammatory signal molecules increased in NASH, as these signaling pathways provide a link between hepatic inflammation and insulin resistance. [25].

It has been recently described the roles of interferon regulatory factors (IRFs), a family of transcription factors that regulate IFN expression, that play important roles in both innate and adaptive immune responses and the potential of IRF regulators in NAFLD treatment, as recently well described in a comprehensive review conducted by Zhang et al. [26].

Additionally, the NLR may also increase through a hormonal mechanism. In 2012, Ahmed and colleagues demonstrated induction of hepatic 11*β*-HSD1 expression and activity following NAFLD progression of worsening hepatic inflammation and injury. [27] 11*β*-HSD1 serves as a primary regulator that catalyzes the reduction reaction of inactive cortisone to active cortisol. [27] As patients progress from steatosis to NASH along the NAFLD spectrum, hepatic glucocorticoid levels are activated and relative hypercortisolemia develops. [27] In response to the relative hypercortisolemia, leukocytosis and lymphopenia are also observed; thus, resulting in a markedly elevated NLR. [17] Therefore, while the current literature is unsure of the primary etiology of NASH, it is clear inflammation plays a central role.

In 2022, Lesmana and colleagues investigated the difference in NLR values among the varying degrees of steatosis and fibrosis against transient elastography (TE) with controlled attenuation parameter (CAP), a gold standard diagnostic tool in steatosis and fibrosis. Patients with mild steatosis had a mean NLR of 1.492 (*p* < 0.001), compared to patients with moderate-severe steatosis with a mean NLR of 2.198 (*p* < 0.001). [14] Patients with nonsignificant fibrosis had a mean NLR of 1.744 (*p* < 0.001), compared to patients with significant fibrosis with a mean NLR of 2.617 (*p* < 0.001). [14] Their data suggest NLR can accurately predict the condition of liver steatosis. [14] As discussed earlier, patients with insulin pathologies including obesity and type 2 diabetes mellitus are at risk of increased NLR alone, without steatosis. The data from Lesmana and colleagues found these comorbidities were not confounding factors in comparing the NLR to CAP as diagnostic tools. [14].

In 2015, Yilmaz and colleagues compared NLR and C-reactive protein (CRP) as variables in understanding liver histopathology and fibrosis. They found NLR to increase significantly with steatosis and fibrosis, whereas CRP did not. [17] Therefore, they concluded NLR to be a noninvasive clinical diagnostic tool for NASH and fibrosis compared to CRP. [17] Yilmaz and colleagues also suggest plasma fasting glucose coupled with NLR to independently predict the severity of the NAFLD activity score, as diabetes can accelerate the pathology of NASH in experimental mouse models. [17, 28].

Some studies have suggested hs-CRP levels to be significantly elevated in patients with NASH versus simple steatosis, as well as in patients with advanced fibrosis. [29, 30] In contrast, Hui and colleagues suggest hs-CRP did not accurately predict the severity of NAFLD from a histological standpoint, as they found no correlation between hs-CRP levels and grades of steatosis, fibrosis, or necroinflammation. [31] Yilmaz and colleagues did find NLR to be associated with both fibrosis and necroinflammation. [17] These data further suggest NLR to be a more powerful predictor of NASH and fibrosis severity.

Our results indicate a significant difference in the predictive value of NLR in NAFLD in retrospective versus prospective studies. We hypothesize this is due to the difference in sample size between the two groups of studies, as we included two retrospective studies versus ten prospective studies. Evaluating more retrospective studies may help clarify the significance of the difference in the predictive value of NLR in the settings of NASH, fibrosis, and NAFLD.

An interesting application may be to evaluate the utility of the NLR applied to alcoholic and pregnancy-related fatty liver disease. One study noted NLR to be significantly higher in both alcoholic liver cirrhosis (ALC) and NAFLD compared to controls. [32] Since inflammatory cell infiltration is the common feature of steatohepatitis in alcoholic liver disease and NAFLD, we expect NLR to also predict histological grade and fibrosis stage in alcoholic liver disease as it seems to do in NAFLD. [33] Furthermore, there do not appear to be any studies evaluating the role of NLR in acute fatty liver of pregnancy (AFLP). Histology of AFLP does not feature inflammation [34, 35], so we predict that NLR may not be able to serve as a marker in this pathology, in contrast to NAFLD and alcoholic liver disease.

### 4.1. Biomarker Usage and Pharmacologic Insights

New biomarkers are important to guide potential treatments as recently reported. In this regard, in the last years. It has been proposed the potential efficacy of sodium-glucose cotransporter 2 (SGLT2) inhibitors on NAFLD and “metabolic associated fatty liver disease (MAFLD) as recently reported by Goya al. [36]. Discussing the potential treatments, also Silymarin can be considered beneficial in treating NAFLD and should be initiated as early as possible and continued as long as necessary as recently suggested by Hashem et al. [37]. Given the results of our study, medications aimed at reducing NLR levels may prove efficacious for treating and even preventing such complications.

### 4.2. Limitations

Our study has a few limitations that are important to address. The main limitation of this study is the small number of papers that were included in the meta-analysis of the association of NLR with liver fibrosis. As such, our results may be limited in power and additional studies would be warranted to further strengthen the results of our study. Furthermore, the studies included in our analysis exhibited high heterogeneity. Although this was accounted for with the random-effect model, such measures may not entirely eliminate the issue of heterogeneity. Nonetheless, our systematic search—in conjunction with a manual review of references from resulting articles—has ensured a thorough and reliable search of the literature and serves as a notable strength of this study.

## 5. Conclusion

In conclusion, the data regarding cirrhotic patients suggest that NLR may be useful as an independent prognostic marker of NASH and liver fibrosis among NAFLD patients. Further studies need to be conducted to determine precise cut-off guidelines in which to utilize NLR.

## Figures and Tables

**Table 1:** General characteristics of included studies.

First author	Year	Country	Design	NASH							Fibrosis							NOS score
				Case		Control		Cut-off point	SEN	SP	Case		Control		Cut-off point	SEN	SP	
				*N*	NLR	*N*	NLR				*N*	NLR	*N*	NLR				
Alkhouri [6]	2011	USA	*P*	50	2.56 ± 1.06	51	1.60 ± 0.61	1.90	72	70	22	2.93 ± 1.49	26	1.73 ± 0.79	2.30	73	77	8
Abdel-razik [5]	2015	Egypt	*P*	120	2.60 ± 1.10	753	1.90 ± 0.70	2.05	73	75	57	2.50 ± 1.10	60	1.80 ± 0.90	2.40	69	79	8
Kara [11]	2015	Turkey	R	179	1.71 ± 0.59	47	1.77 ± 0.58	—	—	—	133	1.76 ± 0.64	93	1.86 ± 0.68	—	—	—	7
Mohamed [15]	2015	Egypt	*P*	30	2.19 ± 0.60	30	1.55 ± 0.36	1.63	77	87	—	—	—	—	—	—	—	6
Yilmaz [17]	2015	Turkey	R	—	—	—	—	—	—	—	9	5.70 ± 0.83	29	2.52 ± 0.86	—	—	—	6
Asil [7]	2016	Turkey	P	15	2.16 ± 0.49	65	1.62 ± 0.43	1.79	87	81	—	—	—	—	—	—	—	7
Kahraman [10]	2016	Turkey	*P*	105	2.12 ± 1.02	38	1.56 ± 0.38	—	—	—	—	—	—	—	—	—	—	7
Chen [8]	2019	China	*P*	22	2.04 ± 0.86	25	1.83 ± 0.66	—	—	—	—	—	—	—	—	—	—	6
Hanafy [9]	2019	Egypt	*P*	201	2.88 ± 0.44	71	1.80 ± 0.50	—	—	—	—	—	—	—	—	—	—	8
Khoury [12]	2019	Israel	R	52	9.70 ± 5.30	39	6.00 ± 3.90	6.60	77	64	56	10.20 ± 4.40	35	4.90 ± 4.40	5.80	88	80	7
Adithya [14]	2021	Indonesia	*P*	70	2.19 ± 0.53	36	1.49 ± 0.30	1.77	82	81	26	2.61 ± 0.51	80	1.74 ± 0.41	2.15	92	88	7
Lauszus [13]	2021	UK	*P*	6	1.72 ± 0.41	14	1.40 ± 0.38	—	—	—	—	—	—	—	—	—	—	6
Jayachandra [16]	2021	India	*P*	43	3.79 ± 1.81	7	2.41 ± 0.79	—	—	—	13	3.27 ± 0.50	13	1.46 ± 0.51	—	—	—	6

NASH = nonalcoholic steatohepatitis; *N *= number; NLR = neutrophil to lymphocyte ratio; NOS = the Newcastle–Ottawa Quality Assessment Scale; *R* = retrospective; *P*: prospective; SEN = sensitivity; SP = specificity.

## Data Availability

The dataset supporting the conclusions of this article is included within the article.

## References

[1] Younossi Z. M., Koenig A. B., Abdelatif D., Fazel Y., Henry L., Wymer M. (2016). Global epidemiology of nonalcoholic fatty liver disease-Meta-analytic assessment of prevalence, incidence, and outcomes. *Hepatology*.

[2] Younossi Z., Anstee Q. M., Marietti M. (2018). Global burden of NAFLD and NASH: trends, predictions, risk factors and prevention. *Nature Reviews Gastroenterology & Hepatology*.

[3] Walsh S. R., Cook E., Goulder F., Justin T., Keeling N. (2005). Neutrophil-lymphocyte ratio as a prognostic factor in colorectal cancer. *Journal of Surgical Oncology*.

[4] Tokgoz S., Kayrak M., Akpinar Z., Seyithanoglu A., Guney F., Yuruten B. (2013). Neutrophil lymphocyte ratio as a predictor of stroke. *Journal of Stroke and Cerebrovascular Diseases*.

[5] Abdel-Razik A., Mousa N., Shabana W. (2016). A novel model using mean platelet volume and neutrophil to lymphocyte ratio as a marker of nonalcoholic steatohepatitis in NAFLD patients: multicentric study. *European Journal of Gastroenterology and Hepatology*.

[6] Alkhouri N., Morris-Stiff G., Campbell C. (2012). Neutrophil to lymphocyte ratio: a new marker for predicting steatohepatitis and fibrosis in patients with nonalcoholic fatty liver disease. *Liver International*.

[7] Asıl M., Dertli R. (2016). The neutrophil-to-lymphocyte ratio as a noninvasive marker in patients with biopsy-proven non-alcoholic steatohepatitis. *Istanbul Medical Journal*.

[8] Chen J., Zheng M., Liu J. (2019). Ratio of conjugated chenodeoxycholic to muricholic acids is associated with severity of nonalcoholic steatohepatitis. *Obesity*.

[9] Hanafy A. S., Seleem W. M., El-kalla F., AbdAlkhalik Basha M., Abd-Elsalam S. (2019). Efficacy of a non-invasive model in predicting the cardiovascular morbidity and histological severity in non-alcoholic fatty liver disease. *Diabetes & Metabolic Syndrome: Clinical Research Reviews*.

[10] Kahraman N. K., Kahraman C., Koçak F. E. (2016). Predictive value of neutrophiltolymphocyte ratio in the severity of non-alcoholic fatty liver disease among type 2 diabetes patients. *Acta Gastro-Enterologica Belgica*.

[11] Kara M., Dogru T., Genc H. (2015). Neutrophil-to-lymphocyte ratio is not a predictor of liver histology in patients with nonalcoholic fatty liver disease. *European Journal of Gastroenterology and Hepatology*.

[12] Khoury T., Mari A., Nseir W., Kadah A., Sbeit W., Mahamid M. (2019). Neutrophil-to-lymphocyte ratio is independently associated with inflammatory activity and fibrosis grade in nonalcoholic fatty liver disease. *European Journal of Gastroenterology and Hepatology*.

[13] Lauszus J. S., Eriksen P. L., Hansen M. M. (2021). Activation and functional priming of blood neutrophils in non-alcoholic fatty liver disease increases in non-alcoholic steatohepatitis. *Clinical and Experimental Gastroenterology*.

[14] Lesmana C. R. A., Kencana Y., Rinaldi I. (2022). Diagnostic value of neutrophil to lymphocyte ratio in non-alcoholic fatty liver disease evaluated using transient elastography (TE) with controlled attenuated parameter (CAP). *Diabetes, Metabolic Syndrome and Obesity: Targets and Therapy*.

[15] Mohamed N. R., Assem M. E. S., Atef A. E. F., Mamdouh A. Y., Mohamed S. E. S., Mosaab S. H. (2015). Neutrophil to lymphocyte ratio as a new marker for predicting steatohepatitis in patients with nonalcoholic fatty liver disease. *International Journal of Advanced Research*.

[16] Jayachandra J., Sree Raksha K. N., Desai R. R., Chetan V., Chandrashekar A. P. (2021). A study on association between neutrophil to lymphocyte ratio and steatohepatitis and fibrosis in patients with non-alcoholic fatty liver disease. *Journal of Evolution of Medical and Dental Sciences*.

[17] Yilmaz H., Yalcin K. S., Namuslu M. (2015). Neutrophil-lymphocyte ratio (NLR) could be better predictor than C-reactive protein (CRP) for liver fibrosis in non-alcoholic steatohepatitis (NASH). *Annals of Clinical Laboratory Science*.

[18] Page M. J., McKenzie J. E., Bossuyt P. M. (2021). The PRISMA 2020 statement: an updated guideline for reporting systematic reviews. *International Journal of Surgery*.

[19] Wells G. A., Shea B. J., O’Connell D. (2000). *The Newcastle-Ottawa Scale (NOS) for Assessing the Quality of Nonrandomised Studies in Meta-Analyses*.

[20] Radulescu D., Baleanu V. D., Padureanu V. (2020). Neutrophil/lymphocyte ratio as predictor of anastomotic leak after gastric cancer surgery. *Diagnostics*.

[21] Fujii H., Kawada N. (2012). Inflammation and fibrogenesis in steatohepatitis. *Journal of Gastroenterology*.

[22] Day C. P. (2002). Pathogenesis of steatohepatitis. *Best Practice & Research Clinical Gastroenterology*.

[23] Kamari Y., Shaish A., Vax E. (2011). Lack of interleukin-1*α* or interleukin-1*β* inhibits transformation of steatosis to steatohepatitis and liver fibrosis in hypercholesterolemic mice. *Journal of Hepatology*.

[24] Farrell G. C., Haczeyni F., Chitturi S. (2018). Pathogenesis of NASH: how metabolic complications of overnutrition favour lipotoxicity and pro-inflammatory fatty liver disease. *Advances in Experimental Medicine & Biology*.

[25] Farrell G. C., Rooyen D. V., Gan L., Chitturi S. (2012). NASH is an inflammatory disorder: pathogenic, prognostic and therapeutic implications. *Gut Liver*.

[26] Zhang C., Liu S., Yang M. (2022). The role of interferon regulatory factors in non-alcoholic fatty liver disease and non-alcoholic steatohepatitis. *Gastroenterology Insights*.

[27] Ahmed A., Rabbitt E., Brady T. (2012). A switch in hepatic cortisol metabolism across the spectrum of non alcoholic fatty liver disease. *PLoS One*.

[28] Ota T., Takamura T., Kurita S. (2007). Insulin resistance accelerates a dietary rat model of nonalcoholic steatohepatitis. *Gastroenterology*.

[29] Yoneda M., Mawatari H., Fujita K. (2007). High-sensitivity C-reactive protein is an independent clinical feature of nonalcoholic steatohepatitis (NASH) and also of the severity of fibrosis in NASH. *Journal of Gastroenterology*.

[30] Targher G. (2006). Relationship between high-sensitivity C-reactive protein levels and liver histology in subjects with non-alcoholic fatty liver disease. *Journal of Hepatology*.

[31] Hui J. M., Farrell G. C., Kench J. G., George J. (2004). High sensitivity C-reactive protein values do not reliably predict the severity of histological changes in NAFLD. *Hepatology*.

[32] Michalak A., Cichoz-Lach H., Guz M. (2020). Towards an evaluation of alcoholic liver cirrhosis and nonalcoholic fatty liver disease patients with hematological scales. *World Journal of Gastroenterology*.

[33] Gao B., Tsukamoto H. (2016). Inflammation in alcoholic and nonalcoholic fatty liver disease: friend or foe?. *Gastroenterology*.

[34] Mabie W. C. (1991). Acute fatty liver of pregnancy. *Critical Care Clinics*.

[35] Ko H. H., Yoshida E. M. (2006). Acute fatty liver of pregnancy. *Canadian Journal of Gastroenterology*.

[36] Goya T., Imoto K., Tashiro S. (2022). The efficacy of tofogliflozin on metabolic dysfunction-associated fatty liver disease. *Gastroenterology Insights*.

[37] Hashem A., Shastri Y., Al Otaibi M. (2021). Expert opinion on the management of Non-alcoholic fatty liver disease (NAFLD) in the Middle east with a focus on the use of silymarin. *Gastroenterology Insights*.

